# Study of the cytotoxicity of asiaticoside on rats and tumour cells

**DOI:** 10.1186/1471-2407-14-220

**Published:** 2014-03-25

**Authors:** Fatma J Al-Saeedi

**Affiliations:** 1 Nuclear Medicine Department, Faculty of Medicine, Kuwait University, Al-Jabriya, Kuwait

**Keywords:** Asiaticoside, DMBA, Tumour, Proliferation, Apoptosis, Rats

## Abstract

**Background:**

Cancer chemoprevention is considered one of the most promising areas in current cancer research, and asiaticoside, which is derived from the plant *Centella asiatica*, has a relative lack of systemic toxicity. The purpose of this study was to investigate whether asiaticoside is effective against 7,12-dimethylbenz(a)anthracene (DMBA)-induced carcinogenicity *in vitro* (MCF-7 and other cells) and *in vivo* (DMBA-induced rat cancer).

**Methods:**

An MTT assay was performed involving the treatment of MCF-7 cells for 48 h with H_2_O_2_ alone and H_2_O_2_ + different asiaticoside concentrations. Flow cytometry was performed, and the level of caspase 3, tumour necrosis factor-alpha (TNF-α) and interleukin-1 (IL-1) were quantified. Adult female Sprague–Dawley (SD) rats were divided into five groups designated I (control), II (DMBA-induced cancer), III (pre- and post-treatment with asiaticoside (200 μg/animal) in DMBA-induced cancer), IV (post-treatment with asiaticoside in DMBA-induced cancer), and V (treated with asiaticoside alone, drug control). Twelve weeks post-DMBA, rats developed mammary tumours. Rats either were sacrificed or imaged with MIBI. Histological examination of tumour tissues was performed. Tumour MIBI uptake ratios were determined. The data are expressed as the means ± standard deviation. Appropriate *t*-test and ANOVA statistical methods were used to compare data.

**Results:**

The IC50 of asiaticoside for MCF-7 cells was determined to be 40 μM. Asiaticoside has potential for hydrogen peroxide cytotoxicity, and the caspase-3 activity increased with increasing asiaticoside dose in MCF-7 cells treated for 48 h. The expression of the cytokines TNF-α and IL-1β was significantly decreased and correlated with MIBI uptake ratios *in vitro and in vivo* after asiaticoside administration.

**Conclusion:**

This study demonstrates that asiaticoside is effective *in vitro* and *in vivo* in inducing apoptosis and enhancing anti-tumour activity.

## Background

According to the American Cancer Society, more than 7.6 million people die from cancer in the world each year [1], and cancer chemoprevention using different drugs and natural agents has been attempted. *Centella asiatica* is a plant that is widely used in traditional Ayurvedic medicine for a variety of illnesses. Recent research has shown that components of *Centella asiatica*, particularly asiaticoside, show great pharmacological effects in the prevention and treatment of cancer [2], ulcers [3] diarrhea, asthma, tuberculosis, various skin lesions, wound healing [4,5], mental disorders [6], and atherosclerosis, as well as a fungicidal antibacterial [7], and antioxidant effects [8,9].

7,12-Dimethylbenz(a)anthracene (DMBA)-induced rat mammary cancer has been widely exploited in cancer studies for many years [10-12]. In this study, we applied our asiaticoside treatment system to a DMBA-induced rat mammary cancer model and human breast cancer (MCF-7) cells.

Technetium-99 m hexakis-2-methoxyisobutylisonitrile, Tc-99 m-sestamibi (^99m^Tc-MIBI) is a cationic lipophilic radiopharmaceutical commonly used in nuclear cardiology that has been reported to be used in several tumour types including those of the breast, lung, thyroid, brain, head and neck, gastrointestinal tract, solid tumours of bones and soft tissues and lymphomas [13-16]. The human breast adenocarcinoma MCF-7 cell line was used because it is a commonly available human breast cancer *in vitro* model. MCF-7 cells have been previously studied using MIBI and have shown high MIBI uptake after 60 min in comparison with other types of cancer cell lines [17-19].

Few studies in the literature investigated the asiaticoside effects on cancer, and the effects of asiaticoside in tumours are limited. In this study, the asiaticoside effects *in vitro* on cancer cells and *in vivo* on DMBA-induced carcinogenesis in rats were investigated via radionuclide imaging and various molecular biology tests.

## Methods

### Materials

MIBI or sestamibi (cardiolite) were purchased from Bristol-Myers Squibb (New York, USA). The pertechnetate (^99m^TcO^-4^) radionuclide was obtained from a molybdenum-99-technetium-99 m (^99^Mo-^99m^Tc) generator purchased from Amersham International plc (Amersham, UK). 7,12-Dimethyl benzanthracene (DMBA), asiaticoside (MW = 959.12) and all other reagents used in this study were supplied by Sigma-Aldrich (UK). Propidium iodide (PI)-ribonuclease (RNase) staining buffer (BD staining kit) was obtained from BD Biosciences.

### The approval of an appropriate ethics committee

All experimental research reported in this manuscript was approved by the Kuwait University Faculty of Medicine scientific local ethics committee.

### Cell culture and media

All of the culture media and supplements were provided by Biowhittaker (Fisher Scientific., Ratastie, Finland, Europe). The human breast cancer MCF-7, MDA-231, pII and HBL-100 cell lines, the prostate cancer PC-3 cell line and the human keratinocyte skin HaCaT cell line were purchased from Cell Lines Service (Eppelheim, Germany) between March and June 2012. MCF-7 cells were grown in advanced Dulbecco’s Modified Eagle Medium (Advanced DMEM) supplemented with 10% foetal calf serum (FCS), 2 mmol/l L-glutamine, 100 units per ml penicillin and 100 mg/ml streptomycin and incubated in a humidified atmosphere with 5% CO_2_: 95% air at 37°C. Unless otherwise stated, stock cultures of MCF-7 cells were seeded at a density of 2 × 10^5^ cells/ml in 25 cm^2^ flasks and allowed to multiply for 48 to 72 h. For chemotherapy experiments, the MCF-7 cells were drug-sensitive/wild type (WT) cells and allowed to grow exponentially to 70% confluency. Cells were cultured in two groups: MCF-7 cells alone (control) and MCF-7 cells treated with different asiaticoside concentrations for 24, 48, or 72 h (treated cells). All cells were tested and authenticated in March 2011 and again tested in June 2012.

### *In vitro* experimental studies

#### Cell viability (MTT) assay

MCF-7 cells (1 × 10^6^) were incubated in 25 cm^2^ flasks in triplicate. The flasks were set up for controls and different asiaticoside concentrations (0.0025, 0.01, 0.02, 0.04, 0.1, 0.2, 0.25, 0.3, 0.5, 1, 10, 20, 40, 50, 125, 250 and 500 μM) and then incubated in a humidified atmosphere with 5% CO_2_: 95% air at 37°C for different time points (24, 48 and 72 h). Measurement of cell viability was determined using the 3-(4–5 dimethylthiozol-2-yl)-2,5 diphenyl-tetrazolium bromide (MTT) assay, which is based on the conversion of MTT to MTT-formazan by mitochondria.

In addition, in some experiments, MCF-7 cells and pII, PC-3, MDA-231 and HBL-100 cells were seeded in flat-bottomed 96-well tissue culture plates in triplicate at a concentration of 1 × 10^5^ cells/ml medium in a volume of 100 μl per well and allowed to grow to 70% confluency before the addition of asiaticoside. After reaching 70% confluency, different concentrations of asiaticoside (0, 0.0025, 0.25, 0.5, 1, 20, 40, and 80 μМ) was separately added and incubated for 24, 48 and 72 h. After the incubation period, the medium was removed, the cells were washed with phosphate buffered saline (PBS), and 100 μl fresh medium was then added together with 20 μl of MTT (5 mg/ml) to each well. The plates were protected from light and incubated for 3 h, and the formazan crystals formed were solubilised with 200 μl dimethyl sulphoxide (DMSO). The plates were maintained in a shaker with gentle mixing for 20 min to dissolve the precipitate. The colour developed was measured in a 96-well plate scanner (Multiskan Spectrum, Thermo Electron Corporation, Vantaa, Finland) at dual filter wavelengths of 540 and 690 nm. The cell viability was expressed as percentage over the control. This viability test was used to determine the optimum asiaticoside inhibitory concentration (IC50) for MCF-7 cells.

#### MTT experiment with hydrogen peroxide (H_2_O_2_)

MCF-7 cells were cultured in 96-well plates (1 × 10^6^ cells per well) in triplicate and then incubated in a humidified atmosphere with 5% CO_2_: 95% air at 37°C. The 96-well plates were prepared for experiments involving H_2_O_2_ alone and different asiaticoside concentrations + H_2_O_2._

For H_2_O_2_ alone, the media was aspirated, the wells were washed with PBS, and different concentrations of H_2_O_2_ diluted in 1% serum media were then added to the wells: 0, 0.025, 0.05, 0.1, 0.2, 0.3, 0.5 and 1 mM.

For H_2_O_2_ + asiaticoside, 1 μM asiaticoside was added and incubated for 2 h. The media was aspirated, a combination of 1 mM H_2_O_2_ and 1 μM asiaticoside was added, and the cells were incubated overnight. After 24 h, the MTT assay was performed as described above.

#### Flow cytometry

A total of 1 × 10^6^ MCF-7 cells/ml were seeded in a 25 cm^2^ tissue culture flask to determine the DNA synthesis phase (S phase) by 2-dimensional (2D) flow cytometry analysis. Cells were treated with different concentrations of asiaticoside e.g., 0, 20, 40, and 80 μМ, and incubated for 48 h. Cells were washed twice in ice-cold PBS, harvested with 0.5% trypsin, and centrifuged. The pellets were resuspended in 5 ml of ice-cold 70% ethanol while vortexing. These resuspended cells were maintained at -20°C overnight. The samples were centrifuged and washed with 2 ml PBS, the samples were centrifuged again and then 1.2 μl RNAase was added. The samples were vortexed and incubated in a water bath at 37°C for 15 min. After the incubation period, 200 μl propidium iodide was added, and the cells were then mixed well and transferred to a FACS tube. Analysis was performed using a Beckman Coulter Cytomics FC 500 (Miami, FL, USA).

#### Assessment of DNA damage

DNA damage was assessed using the cell-death detection ELISA procedure described below.

#### Cell death detection, enzyme-linked immunosorbent assay (ELISA) procedure

MCF-7 cells were cultured in 6-well plates (2 × 10^5^ cells per well) in triplicate and washed with PBS; different concentrations of asiaticoside dissolved in 1% serum media (0, 50, 100 and 200 μМ) was added, and then the cells were incubated for 48 h.

A total of 100 μl coating solution was added into each well of the MP-module and incubated for 1 h at 15–25°C. The coating solution was thoroughly removed by tapping or suctioning. A total of 200 μl incubation buffer was added into each well of the MP-module and incubated for 30 min at 15–25°C. The wells were washed with 250–300 μl per well washing solution 3 times, and the washing solution was carefully removed. A total of 100 μl of sample solution was added into each well of the MP-module. The solution was thoroughly removed by tapping or suctioning. The wells were washed with 250–300 μl per well washing solution 3 times, and the washing solution was carefully removed. A total of 100 μl of substrate solution was added into each well of the MP-module and incubated on a plate shaker at 250 rpm until the colour development was sufficient for photometric analysis (approximately 10–20 min). The contents of the wells were then homogenised by careful tapping at the MP-module edges, and the plates were measured at 405 nm against substrate solution as blank.

#### Apoptosis

MCF-7 cells were plated in 6-well plates (1×10^6^ cells/well) overnight. The following day, the cells were washed with PBS, and 1 ml RPMI media without foetal bovine serum (FBS) was added and incubated overnight. Different concentrations of asiaticoside (0, 0.5, 1, 50, 125, 250, 500 μM) were added and incubated for a 24, 48, or 72 h incubation period. Apoptosis assays were performed by trypsinising MCF-7 cells and centrifuging the cells together with the aspirated media. Cell pellets were washed twice with ice-cold PBS. A total of 10 μl Annexin V-PE and 100 μl 1X binding buffer was added to the pellet in a 15 ml centrifuge tube and incubated for 15 min on ice in the dark. After incubation, 10 μl 7AAD was added with 380 μl 1X binding buffer, making the total volume of 500 μl. The ‘0’ concentration (control) was labelled as reference, and 500 μl 1X binding buffer was added without Annexin V-PE and 7AAD. All content in the tubes was transferred to 10 ml glass tubes, and an apoptotic assay was then performed using flow cytometry (Beckman Coulter Cytomics FC 500, France).

#### Apoptosis markers

The levels of caspases 3 and 9 were estimated in treated MCF-7 cells in comparison with controls. The levels of p53, NF-kB, phosphoinositide-3 kinase (PI3K), Bcl2 and Bcl2 family proteins (Bax, Bak, Bad, Bcl-Xs, Bid, Bik, Bim and Hrk) were determined and assayed. TRAIL receptors-1 and 2 (TRAIL-R1 and TRAIL-R2), death receptors 3, 4, and 5 (DR3, DR4 and DR5) and tumour necrosis factor (TNF) superfamily Fas associated death domain were quantified by western blot analysis. This analysis was performed to determine the pathway through which the cells undergo apoptosis. The determination of cell cycle phase by flow cytometry was to determine whether the cells were undergoing apoptosis.

#### Western blot

MCF-7 cells were cultured in 25 cm flasks and incubated in a humidified atmosphere with 5% CO_2_: 95% air at 37°C until reaching 70% confluency. The plates were set up in triplicate for controls and asiaticoside-treated cells. The asiaticoside concentration used was the determined IC50 (40 μM), and the cells were incubated for 48 h. After incubation, the cells were gently resuspended in 75 μL RIPA buffer and incubated on ice for 30 min. The cells were then centrifuged for 10 min at 10,000 × *g* and 4°C. Supernatant representing the total cell lysate was maintained at -80°C. The protein concentration of the sample was determined with an Ultrospec 2100 pro UV/Visible Spectrophotometer (GE Healthcare, USA).

The extracted protein was quantified with a BCA protein assay. Protein levels were evaluated by densitometry using a GS-800 Calibrated Imaging Densitometer (Bio-Rad Laboratories, USA).

Protein samples (30 μg) were resolved in a 12% sodium dodecyl sulphate polyacrylamide gel electrophoresis (SDS-PAGE) gel at 100 V for 90 min. The samples were then placed in a 3% 50 ml blocking solution in a clean petri dish for 1 h with gentle shaking. After blocking, the membrane was washed in PBS and probed with a primary antibody for 1 h at room temperature. After incubation, the membrane was washed 3 times with a 20% Tween 20 1× PBS solution and then probed with a secondary antibody for 1 h with shaking, then placed in a developing solution.

#### Caspase-3 fluorescence ELISA assay

MCF-7 cells (1 × 10^6^ cells/well) in 2 ml of culture medium were seeded in 6-well plates in triplicate. The following day, the cells were treated with different concentrations of asiaticoside, 0, 50, 100, and 200 μМ, and incubated at 37°C for 48 h. The plates were centrifuged at 800 × *g* for 5 min, the culture medium was aspirated and 200 μl of caspase-3 assay buffer was added to each well. A total of 100 μl cell based assay lysis buffer was added to each well, and the plates were incubated with gentle shaking on an orbital shaker for 30 min at room temperature. The plates were centrifuged at 800 × *g* for 10 min, and 90 μl of the supernatant from each well was transferred to a corresponding well in a new black 96-well plate. Next, 10 μl of caspase-3 inhibitor solution was added to the appropriate wells in the black plate, and 100 μl of active caspase-3 standard was also added to wells in the same Plate. A total of 100 μl caspase-3 substrate solution was added to each well, the plates were incubated at 37°C for 30 min and the fluorescent intensity of each well was simultaneously measured at an excitation window of 485 to 535 nm.

#### Immunostimulation effects of asiaticoside

The immunostimulation of the asiaticoside was tested in cancer-induced rats. The mRNA expression of the complement components platelet activating factor (PAF), cyclooxygenases 1 and 2 (COX1 and COX2), tumour necrosis factor-alpha (TNF-α) and interleukin-1 (IL-1) was studied using reverse transcription-polymerase chain reaction (RT-PCR). This assay helps to determine whether asiaticoside mediates or inhibits inflammation.

#### RNA extraction and real-time PCR (RT-PCR)

A total of 500 μl TRIzol reagent was added to frozen cell pellets or tissue samples; these mixtures were homogenised, and 100 μl chloroform was added. The RNA remained exclusively in the aqueous phase. The aqueous phase was transferred into a fresh tube, and the RNA from the aqueous phase was precipitated by mixing with 500 μl isopropanol and incubating at -20°C overnight. The RNA pellet was washed twice with 500 μl cold 70% ethanol, was dissolved in 25 μl RNAse-free water and incubated for 10 min at 60°C. The purity and yield of the RNA was quantified by measuring the absorbance of the RNA solution at 260 and 280 nm.

The extracted RNA was normalised to 500 ng and converted to cDNA using the high capacity cDNA reverse transcription kit (Applied Biosystems, California, USA). DNase treatment of RNA samples prior to RT-PCR was performed according to the manufacturer’s instructions. RT-PCR for the converted cDNA was performed using the Taqman Fast reagent starter kit (Applied Biosystems, California, USA) with an Applied Biosystems 7500 Real-Time PCR System (Applied Biosystems, California, USA). Global gene expression analysis was performed (data not shown), and some well-known candidate genes were selected and investigated, including Bcl2 and Bcl2 family proteins (Bax, Bak, Bad, Bcl-Xs, Bid, Bik, Bim and Hrk), COX-1, COX-2, IL-1, and TNF-α.

### *In vivo* experimental studies

#### Experimental animals

Adult female Sprague–Dawley (SD) rats (250 ± 50 g body weight, 8 weeks age) bred at the Animal Facility of the Faculty of Medicine, Kuwait University, were used in this study (total n = 20). The animals had free access to water and food and were housed 4–5 rats per cage and maintained at 23 ± 2°C in a 12 h light:dark cycle. The animals were handled in accordance with an established animal use protocol following the recommendations of the Kuwait University’s institutional animal care and use committee (IACUC).

#### Experimental protocol

**Figure 1:**
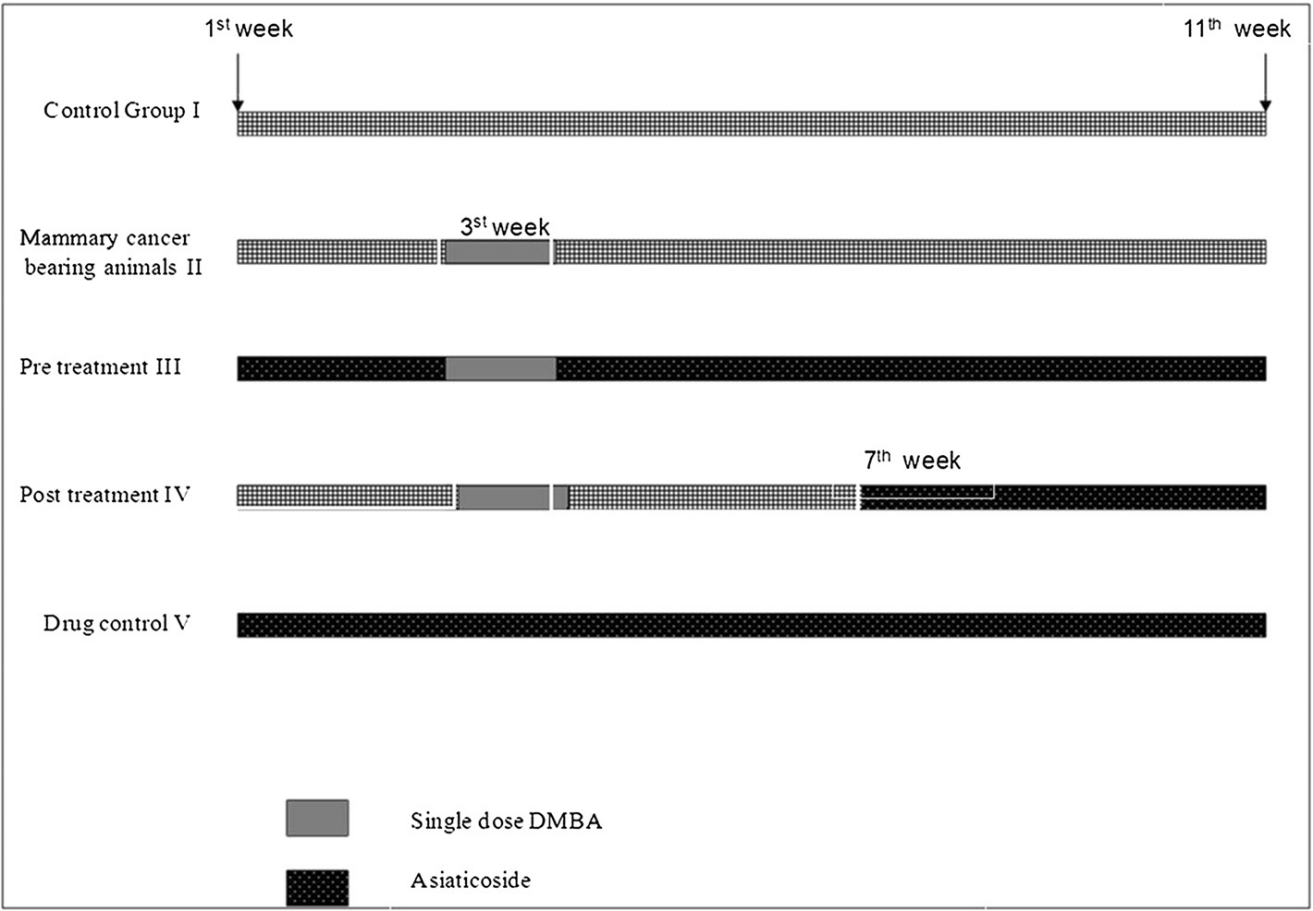
The experimental protocol.

SD rats were randomly divided into five different groups (Figure 1), and experiments were performed accordingly with each consisting of 15 inbred SD rats designated I, II, III, IV, and V. Group I served as normal control animals and were given sesame oil from the 1^st^ to 11^th^ week via oesophageal intubation. Group II represented tumour-bearing rats. In this group, tumours were induced in the 3^rd^ week by a single dose of 0.5 ml DMBA (10 mg/animal) in sesame oil administered via oesophageal intubation, and the rats were given only oil (1–2 and 4–11 weeks) in the subsequent weeks. Group III animals were subjected to pre- and post- treatment with asiaticoside (200 μg/animal) given intraperitoneally (ip) at 1–2 and 4–11 weeks each twice, and tumours were induced in the 3^rd^ week. Group IV animals were treated with a similar dose of asiaticoside from week 7, and DMBA was induced in the 3^rd^ week. Group V animals were treated with asiaticoside alone (drug control). Animals were palpated weekly for tumour formation. At 12 weeks post-DMBA exposure, all animals were sacrificed. At this time, all tumours, skin and visceral organs were saved by snap freezing in liquid nitrogen and then stored at -80°C for further molecular analysis.

#### Weight gain and tumour development

The animal body weight in grams was recorded every three weeks. The weight homogeneity index (HI) was calculated at the beginning of the study according to the formula HI = W_l_/(W_l_ + W_h_)/2, where W_l_ is the lowest weight and W_h_ is the highest weight found in all groups. In addition, body weight gain (W_g_) in grams was calculated according to the formula W_g_ = (W_x_-W_0_)/W_0_*100, which considers the weight recorded in the beginning (W_0_) and at the end (W_x_) of the study. All animals were monitored for tumour development. The tumour mass was measured horizontally and vertically using a calliper. The tumour volume (V) was calculated according to the formula V = (a(b)^2^)/2, where ‘a’ and ‘b’ are the longest and shortest diameters of the tumour, respectively, as described in Carlsson et al. [20].

#### Preparation of ^99m^Tc-MIBI (MIBI)

Lyophilised MIBI vial products were reconstituted using 1110 MBq of fresh ^99m^TcO^-4^. The vials were heated in a boiling water bath for 10 min. Quality control procedures were performed according to the manufacturer’s instructions after cooling in room temperature using Whatman-1 paper and chloroform:methanol solution (75:25). An MIBI labelling efficiency greater than 95% was used.

#### MIBI tumour uptake imaging and processing

The rats in each group were anaesthetised using an intraperitoneal (ip) injection of ketamine: xylazine (40 mg/kg: 5 mg/kg body weight; Serumwerk, Bernburg, Germany) injected with 37 MBq MIBI and imaged in two dynamic phases: a vascular phase at 1 sec/frame for 1 min and a subsequent parenchymal phase at 1 min/frame for 1 h.

Images were visualised in a 0–61 min composite image. The regional distribution of MIBI was determined by drawing a region of interest (ROI) over tumours and other vital organs such as the heart, liver, spleen, bladder and whole body (WB). The tumour to WB ratio for MIBI before DMBA (control) and after DMBA or asiaticoside administration was obtained.

#### Apoptosis markers and apoptosis analysis by flow cytometry and RT-PCR

These assays were performed as described above *in vivo* in rats with DMBA-induced mammary carcinogenesis treated with asiaticoside in comparison with controls.

#### Presentation of data and statistical analysis

Unless otherwise stated, all data are expressed as the means ± standard deviation (means ± SD). Student’s *t*-test was used to determine significant differences between two means, while Kruskal-Wallis non-parametric analysis of the one-way analysis of variance (ANOVA) test was used to evaluate differences between study groups. Statistical analysis was performed using SPSS version 17.0 software (Chicago, USA).

## Results

### Cell viability (MTT) assay

**Figure 2:**
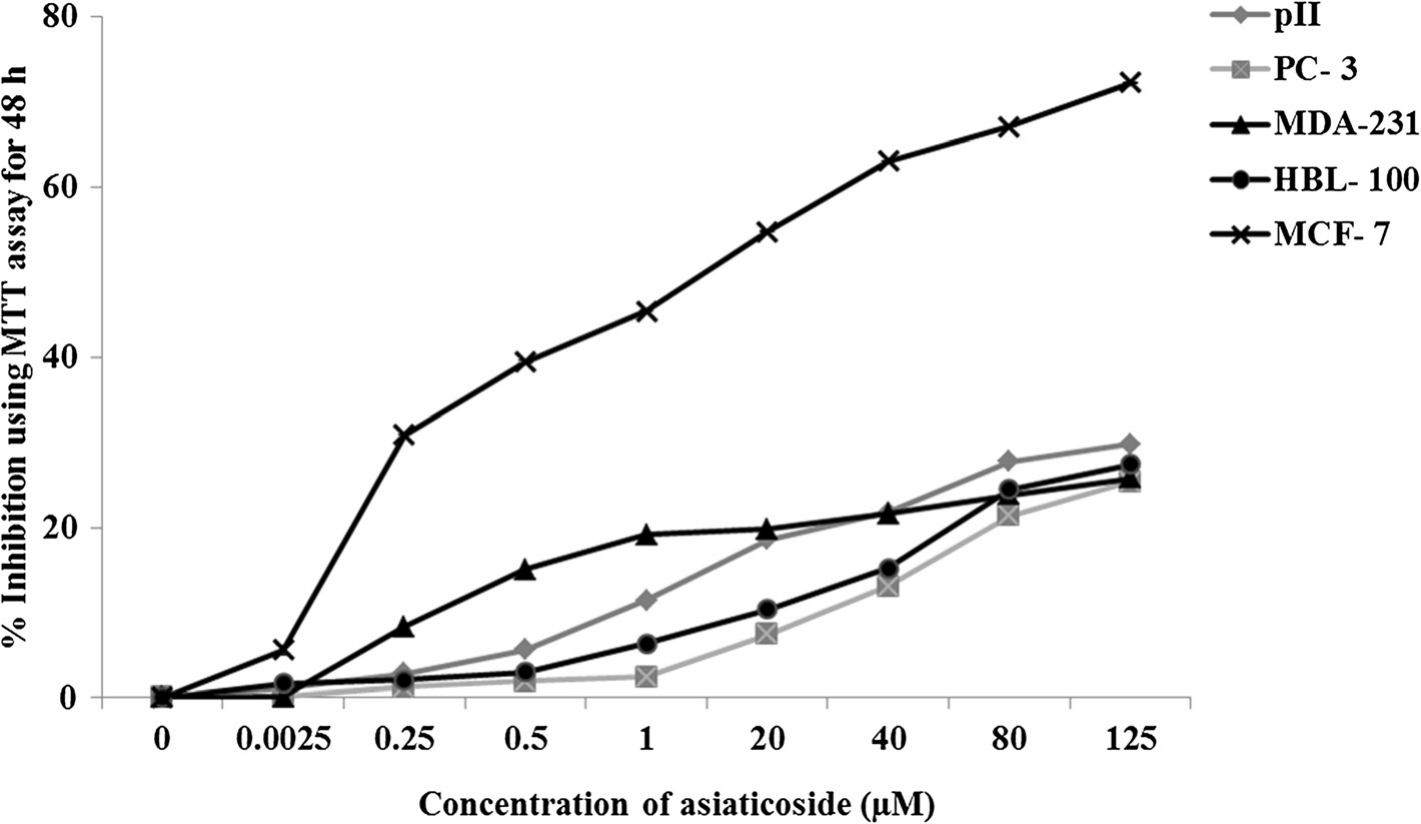
Raw data for the percentage of cell inhibition for a 48 h asiaticoside incubation period, showing no effects of asiaticoside on cell lines with the exception of the MCF-7 cell line upon MTT assay.

The viability, which was expressed as the percentage of inhibition over control for MCF-7 cells for 24, 48, and 72 h, was obtained with an MTT assay. The IC50 value of asiaticoside for MCF-7 cells was detected, and it was determined to be 40 μM at 48 h. As described above in the Methods section, experiments involved treatment with different concentrations of asiaticoside for 24, 48, and 72 h in different cell lines (MCF-7, MDA-231 and HBL-100, PC-3 and pII) using the MTT assay. The results demonstrated that asiaticoside had no effect on the cell lines at these concentrations with the exception of the MCF-7 cells (Figure 2).

### MTT experiments with H2O2

**Figure 3:**
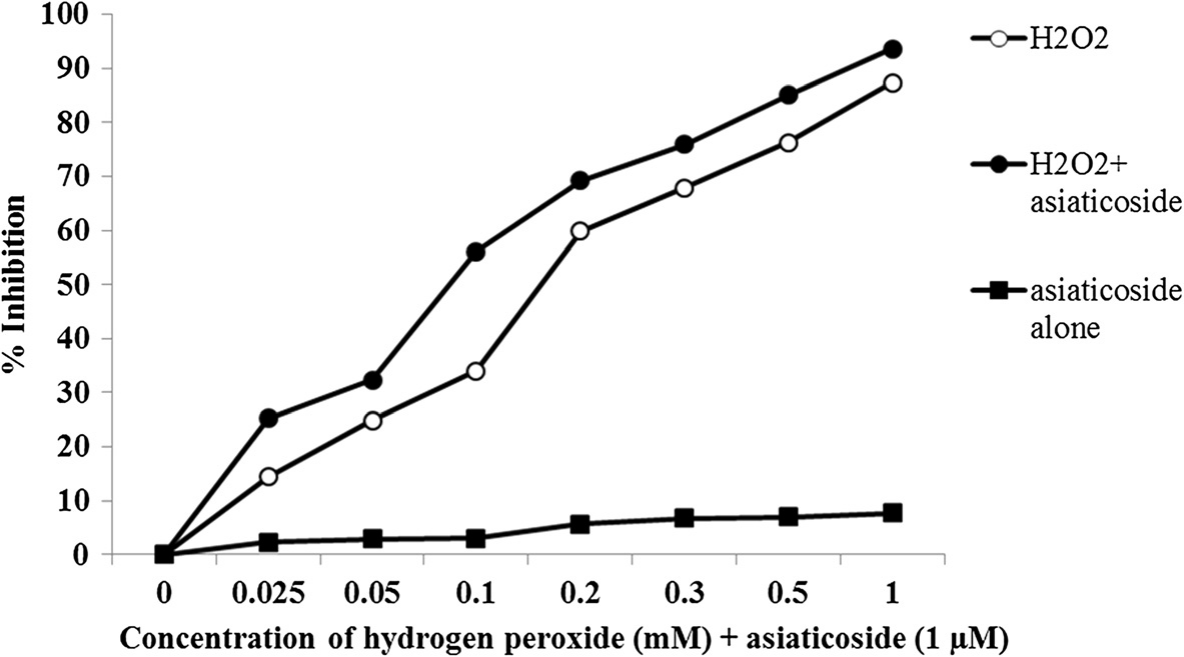
The percentage of asiaticoside cell inhibition over control for a 48 h incubation period and the potential for hydrogen peroxide cytotoxicity using an MTT assay for MCF-7 cells.

Figure 3 shows the percentage of cell inhibition over control for a 48 h incubation period and the asiaticoside potential for the cytotoxicity of hydrogen peroxide using an MTT assay with MCF-7 cells. The use of H2O2 in the MTT experiment was to induce oxidative stress, apoptosis and cytotoxicity. Asiaticoside does not cause genotoxic effects in cell lines.

### Assessment of DNA damage, cell death detection, ELISA and caspase-3 fluorescence

**Figure 4:**
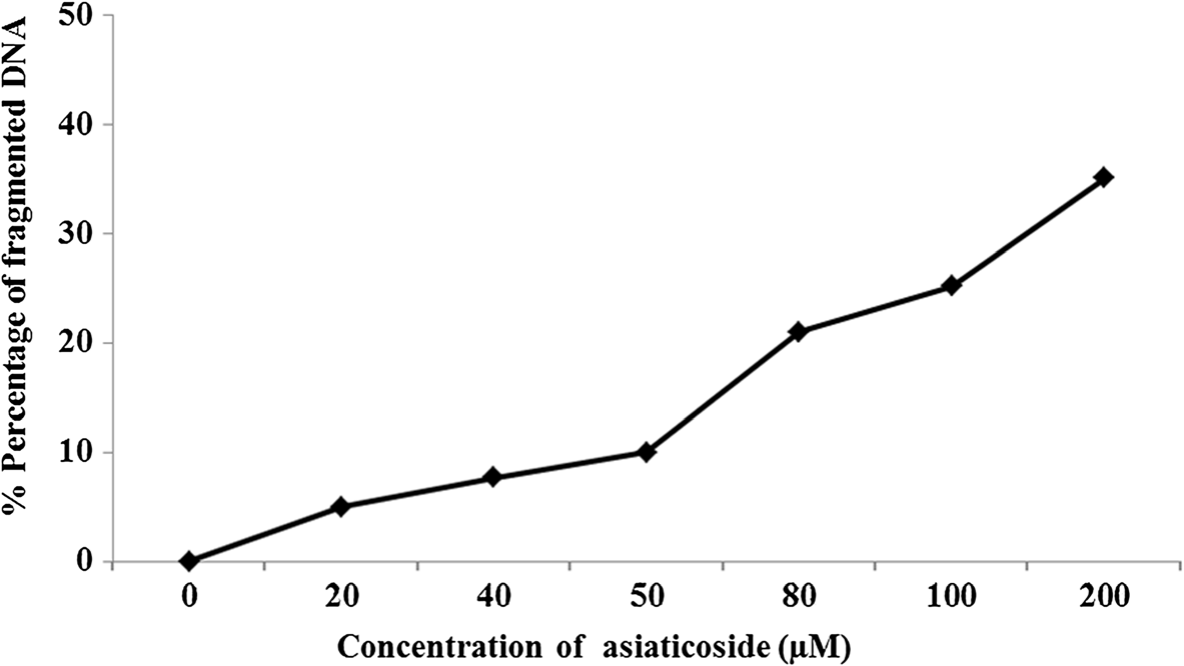
Percentage of fragmented DNA in response to the effects of asiaticoside.

**Figure 5:**
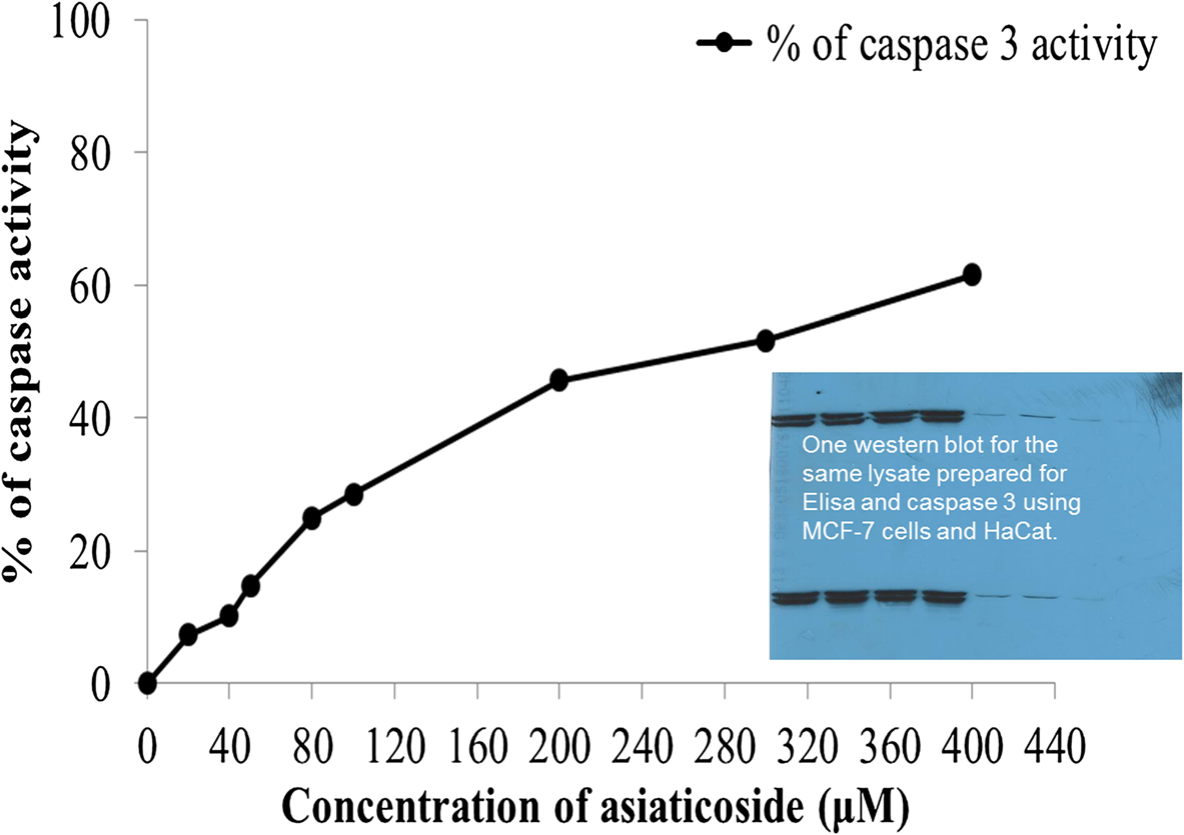
**Percentage of fragmented DNA in response to the effects of asiaticoside using a caspase-3 fluorescence assay.** To the right of the graph is shown a western blot for the same lysate prepared for ELISA and caspase 3 assays using MCF-7 and HaCaT cells.

Figure 4 shows the percentage of fragmented DNA and cell death in response to the effects of asiaticoside administration in MCF-7 cells as detected by ELISA. The percentage of cell death (inhibition) increases with asiaticoside concentration. In addition, cell death was detected using a caspase-3 fluorescence assay. Asiaticoside downregulated the expression and activity of caspase-3 (Figure 5).

### DNA synthesis and apoptosis marker measurement by flow cytometry

**Figure 6:**
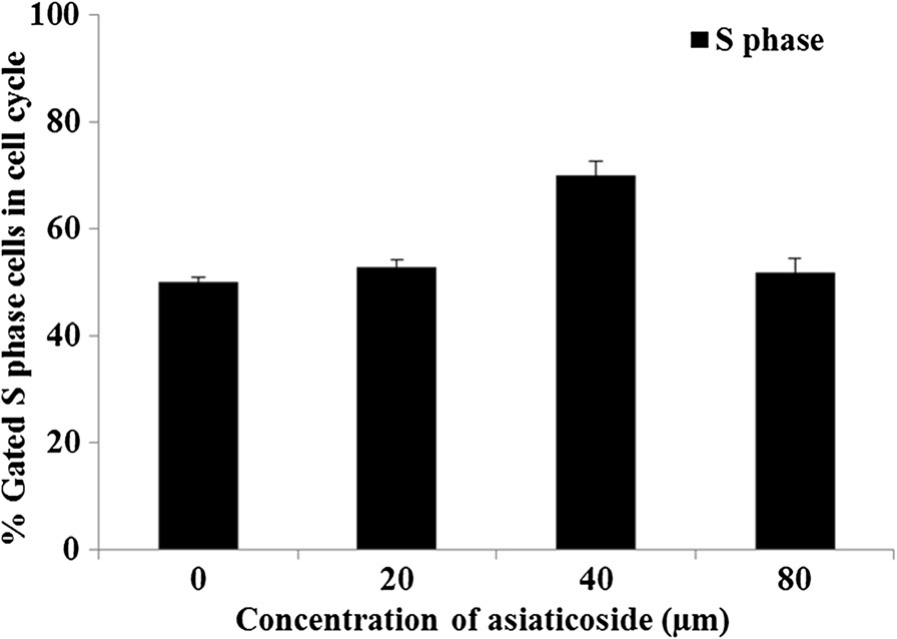
Histograms of DNA synthesis with 0, 20, 40 and 80 μm asiaticoside treatment (S phase; % gated S phase cells in cell cycle) as measured by flow cytometry.

DNA synthesis (S phase) and apoptosis were measured by flow cytometry. Histograms were generated to determine the cell cycle phase distribution after debris exclusion. The sub-G_1_/G_0_ peaks were considered to be representative of apoptotic cells, and the S histogram bars were representative of DNA synthesis phase cells. S phase values were highly significantly higher at all concentrations (0, 20, 40, and 80 μm asiaticoside) compared with other cell cycle phases, and there were significant differences at 40 μm as compared with other concentrations and control (Figure 6).

### Determination of *in vitro* and *in vivo* mRNA expression by RT-PCR

**Figure 7:**
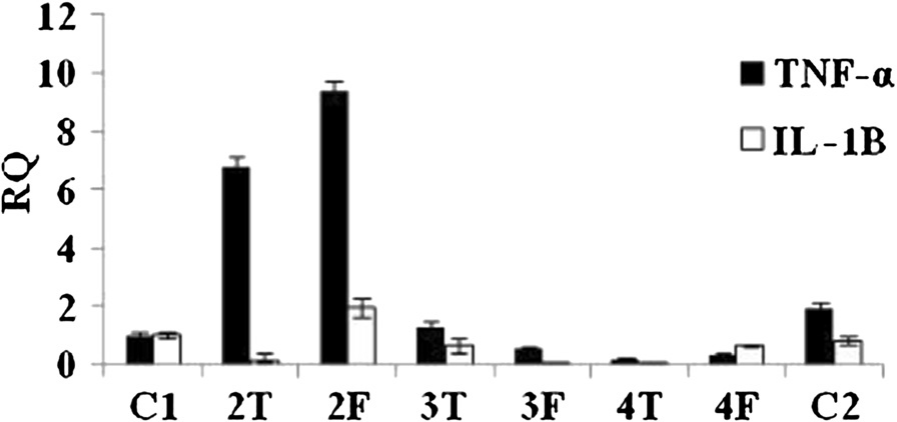
**Cytokinin, necrosis factor-alpha (TNF-α) and interleukin-1 beta (IL-1β) expression in DMBA-bearing tumours after asiaticoside administration as determined by RT-PCR.** C: tumours in thoracic chest and F: femoral site.

The results showed that asiaticoside has an effect on cytokinin expression in DMBA-bearing tumours (Figure 7) as well as in MCF-7 cells. Asiaticoside led to decreased tumour necrosis factor-alpha (TNF-α) and interleukin-1 beta (IL-1β) expression. Asiaticoside affected neither pro-apoptotic Bax nor anti-apoptotic Bcl-2 expression.

### *In vivo* animal imaging

#### Weight gain and tumour development

**Figure 8:**
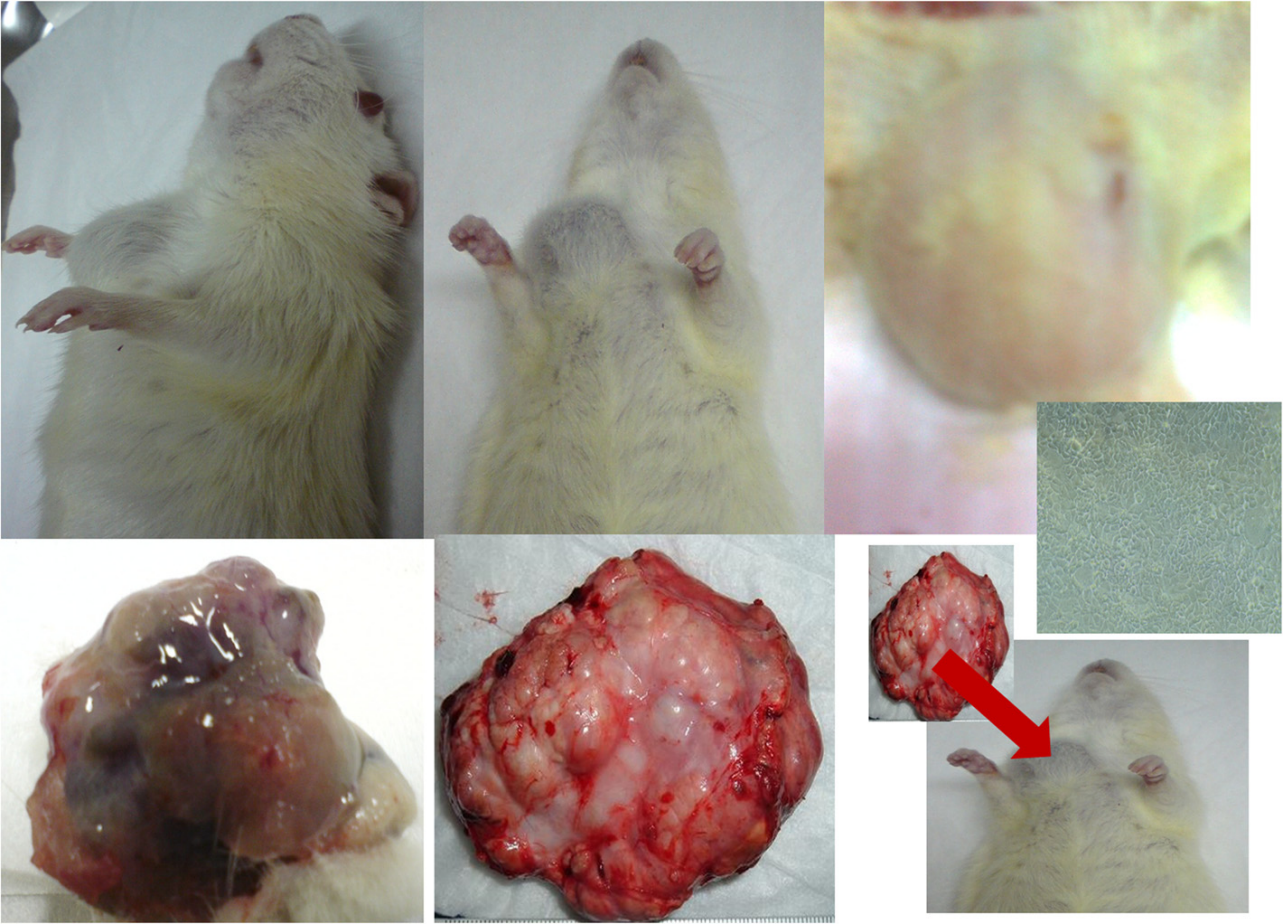
**Figure presenting images of breast cancer tissue used for *in vivo *** **study.**

Animals from each group were healthy, and after the 8^th^ week of experiments, tumours were mostly observed in groups II, III, and IV. The developed tumours were soft, rubbery and more adherent to the skin than the body wall (Figure 8). In 15 rats, multiple tumour growths were observed at 1–4 sites in the body, including tumours in the left and right thoracic chest (C) and the femoral site (F) for groups II, III, and IV (rats receiving DMBA). Without treatment, DMBA-induced tumours grew rapidly but did not metastasise.

The weight of the animals for all groups was similar as indicated by the homogeneity index. The animal body weight (mean gain ± SD) in grams was determined every 3 weeks and found to be increased during the study (Table 1). The mean weight progressively increased during the study.

**Table 1:** The means of the animal body weight and gain ± SD during the experiment for each group in grams

**Week**	**Group I**	**Group II**	**Group III**	**Group IV**	**Group V**
1	251 ± 50	250 ± 50	254 ± 50	251 ± 50	254 ± 50
3	285 ± 10	282 ± 10	284 ± 10	282 ± 10	286 ± 10
6	318 ± 10	305 ± 10	308 ± 10	302 ± 10	318 ± 10
9	349 ± 10	332 ± 10	335 ± 10	336 ± 10	350 ± 10
12	395 ± 10	379 ± 10	384 ± 10	382 ± 10	392 ± 10

After 12 weeks of the study, groups II, III, and IV (rats receiving DMBA) showed 5.8, 6.2, and 5.2% less body weight compared with group I. In other words, rats not receiving DMBA (controls) were 5-7% heavier than rats receiving DMBA. No significant difference (p < 0.05) was found between group I (control) and group II (tumour bearing rats).

**Figure 9:**
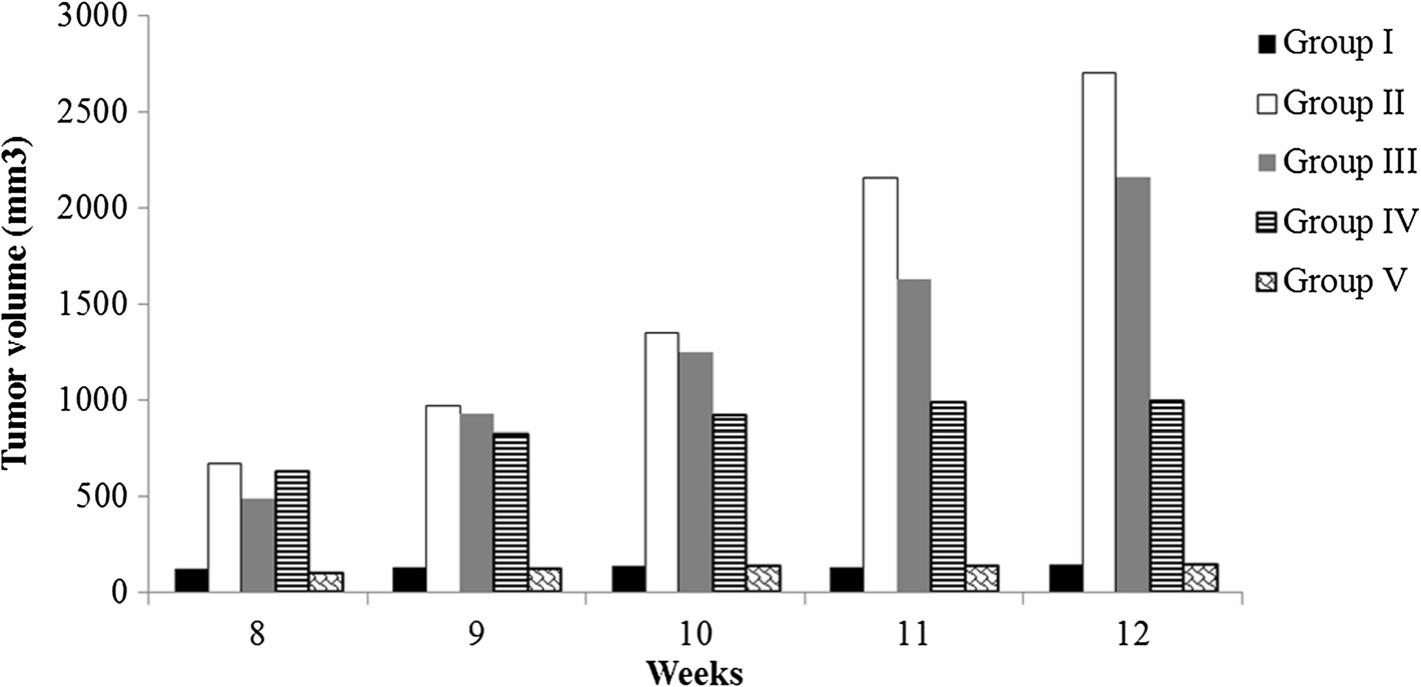
**Weekly animal tumour growth data (volume in mm**^**3**^**) for all 5 groups beginning with the 8**^**th **^**week of study.**

Results showed a tumour volume increase on a weekly basis beginning with the 8^th^ week, which is presented in graphic form in Figure 9. The tumour volume was calculated by multiplying the length of the tumour by the square of the width and dividing the product by two. To obtain statistically significant results, the experimental groups were repeated.

The results showed that rat tumours from groups III and IV (treated with asiaticoside) showed significant less tumour progression (p < 0.001) as compared to group II (positive control, Figure 9).

In addition, rat tumours from groups II, III, and IV were grounded into powder to perform further studies. RNA and protein extraction was then performed according to the procedure described above. Western blotting and RT-PCR was performed from these samples.

#### MIBI radiotracer *in vitro* uptake determination

In some experiments, *in vitro* MIBI uptake in MCF-7 cells was detected as described in Al-Saeedi et al. [17]. The uptake results were expressed as radioactivity in MBq/mg of protein. In the control (no asiaticoside, 0) and at 10, 20, 30, 40, and 50 μM asiaticoside, the mean ± SE levels of MIBI uptake were 0.95 ± 0.007, 0.81 ± 0.009, 0.79 ± 0.019, 0.63 ± 0.004, 0.13 ± 0.006 and 0.07 ± 0.008, respectively. The uptake was dose dependent, and asiaticoside inhibited 47% of MIBI uptake. A significant reduction in MCF-7 cell uptake was observed at concentrations higher than 20 μM asiaticoside with a p = 0.03 using Student’s paired *t* test compared with the control.

#### MIBI tumour uptake imaging and processing

**Figure 10:**
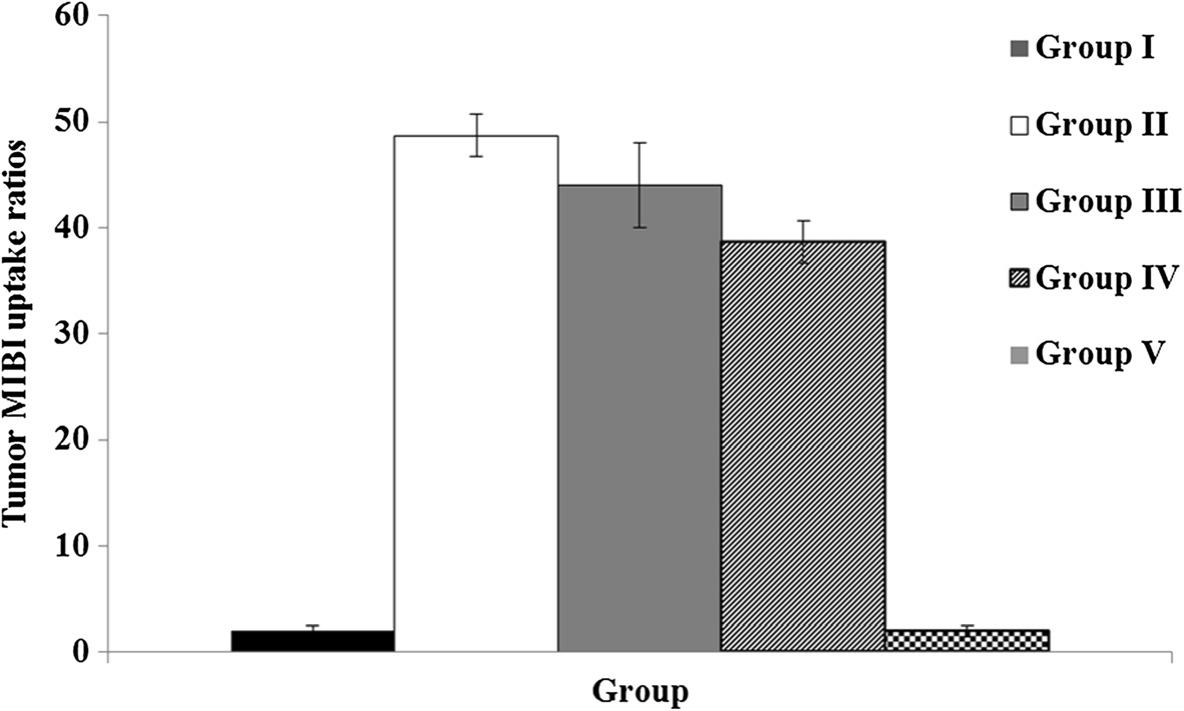
Histograms of the tumour MIBI uptake ratio of all groups expressed as the means ± SD.

The regional distribution of MIBI uptake count in tumours was determined and divided by the whole body count in groups II, III and IV, to obtain tumour MIBI uptake ratios. The results showed that the administration of asiaticoside (200 μg/animal) significantly reduced tumour MIBI uptake ratios (p = 0.026) using Student’s paired *t* test. Figure 10 shows histograms of the tumour MIBI uptake ratio for all groups expressed as the means ± SD.

**Figure 11:**
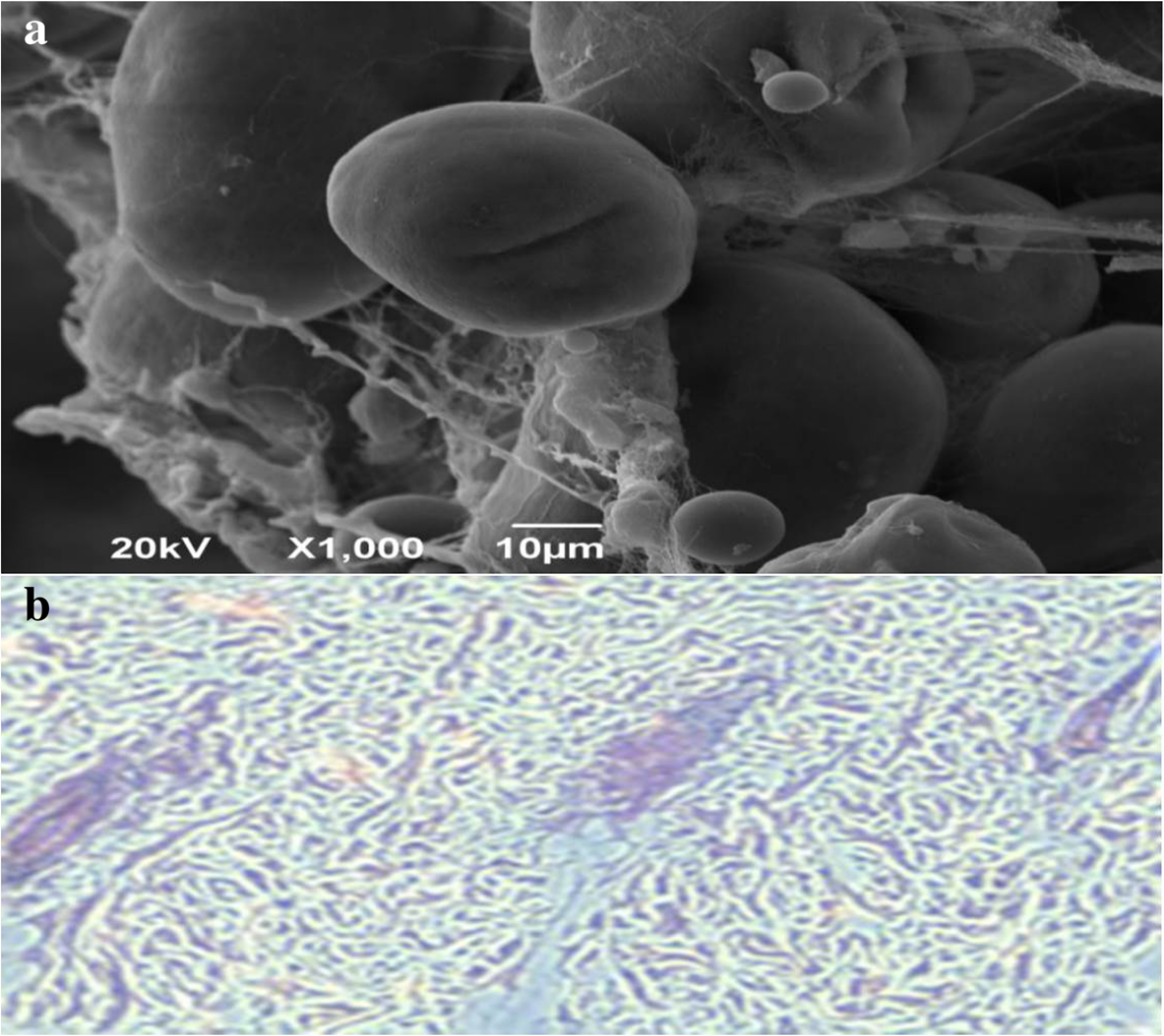
**A histological section of developed tumour 12 weeks post-DMBA exposure, a: tumour scan using scanning electron microscopy (SEM) imaging. b**: tumour scan using trichrome staining.

Scanning electron microscopy (SEM) was performed to investigate changes in animal groups. Preliminary results indicated that all organs were normal, and there was no difference between them. The only differences were observed for groups II, III, and IV, which showed tumours that manifested as basal cell carcinomas. Tumours attached to the skin show that most of the connective tissue transformed into tumour cells 12 weeks post-DMBA exposure upon SEM imaging (Figure 11a). The tumours were histologically adenocarcinomas without the tendency to develop metastases. Individual tumour cells have rounded nuclei, moderate pleomorphism, coarse chromatin, inconspicuous nucleoli, a moderate amount of eosinophilic cytoplasm, and rare mitotic figures. Figure 11b shows tumours with trichrome staining.

The results also showed that administration of 200 μg asiaticoside/animal significantly reduced the percent of tumour growth compared with the control (p = 0.001) using Student’s paired *t* test. The percent of tumour growth (mean ± SD) was as follows: 32.85 ± 2.0 in group II, 23.30 ± 2.0 in group III and 12.85 ± 2.0 in group IV.

## Discussion

In this study, the IC50 of asiaticoside in MCF-7 cells was determined. In addition, the asiaticoside potential for the cytotoxicity of hydrogen peroxide and the percentage of fragmented DNA in MCF-7 cells was detected. Our results are in agreement with studies that have investigated the effects of asiaticoside in MCF-7 cells [17,21]. This finding suggests that asiaticoside stimulates the process of programmed cell death, apoptosis, by a certain mechanism. For the mechanism of asiaticoside, Gurfinkel et al. reported that disruption of the cellular endoplasmic reticulum and alterations in calcium homeostasis are early events in asiaticoside-induced apoptosis [22]. Another study suggested that asiaticoside administration causes a disturbance in mitochondrial function [23] as manifested by apoptosis [2].

Our results showed an increase in the activity of caspase 3 and S phase, which is in agreement with a study that reported the anti-tumour effects of asiaticoside involving activated caspase-3 protein. Asiaticoside plus vincristine enhanced S-G(2)/M arrest, up-regulated cyclin B1 protein expression, and down-regulated P34(cdc2) protein expression in KB cells [21].

Many studies have reported that the Sprague–Dawley (SD) rat animal model can lead to promising conclusions for chemoprevention [24-29]. In this study, we used SD rats as our model. This study showed that DMBA induced tumours in female SD rats beginning with the 8^th^ week in our experiments. This observation is in agreement with earlier studies that have reported that the administration of polycyclic hydrocarbon 7,12-dimethylbenz(a)anthracene (DMBA) to female SD rats at day 50 produces primary mammary carcinomas in all animals within 2 to 3 months [21,30].

Tumours were observed in groups II, III, and IV in our study. These tumours were palpable and observed visually. In addition, histopathology using light and scanning electron microscopy was performed to confirm the development spontaneous tumour tissue and its type. No metastasis was found in other organs. This result is in agreement with many studies. Human breast cancer usually originates in the ductal region, and here, the DMBA-induced mammary tumour model exhibits the same origin [31-34].

In our study, the body weight of the rats did not show significant differences between normal and tumour-bearing rats, suggesting that our experimental model did not produce side-effects that could cause weight loss. In contrast, in studies by Perumal et al. [35] and Padmavathi et al. [36], although there was no initial significant change in body weight for the control and experimental rats, there was a significant (p < 0.001) decrease in body weight for DMBA-induced tumours in female SD rats at the end of the study.

In addition, our results are in agreement with studies that have investigated the effects of *Centella asiatica* (aqueous extract) and asiaticoside in protecting against the adverse effects of radiation (gamma-irradiation). *Centella asiatica* rendered significant radioprotection against radiation-induced body weight loss [37,38].

MIBI is an accurate and efficient test for the detection of breast malignancies [39,40]. MIBI was used to determine tumour uptake and *in vivo* functional imaging by normalising with whole-body region counts. Asiaticoside treatment reduced the tumour uptake of experimental rats.

Here, we demonstrated that after a course of asiaticoside treatment, all rats that developed multiple mammary tumours exhibited tumour regression and a reduction in MIBI uptake. This result is in agreement with a study by Al-Saeedi et al. [17], who demonstrated that *in vitro* MIBI uptake in MCF-7 breast cancer cells was dose dependent and that asiaticoside significantly inhibited 47% of MIBI uptake in comparison with control. MIBI uptake significantly decreased with increasing asiaticoside concentration. This observation suggests that asiaticoside stimulates apoptosis by a certain mechanism. Several studies reported that asiaticoside acts as a biochemical modulator, and it may induce apoptosis or have protective effects such as against beta-amyloid neurotoxicity [41].

MIBI is associated with mitochondrial integrity and cellular viability [42]. Oxidative stress is induced in a cancer-bearing rat, and asiaticoside may induce chemopreventive action against cancer on a molecular mechanism basis.

Scintimammography using MIBI was proposed recently by Khalkhali et al. [43,44] and other investigators for the detection of breast cancer. The sensitivity in this study was found to be up to 94% with a specificity of 88%.

Abdel-Dayem et al. [45] reviewed the intracellular uptake of MIBI and found that in contrast with Tl-201 m chloride, MIBI does not concentrate in inflammatory lesions despite the fact that these lesions are known to have a high rate of metabolism. These authors summarised a few studies and found that the entry of this complex is accomplished by the combination of charge and lipophilicity. The retention of MIBI, which occurs in the mitochondria, is related to the rate of mitochondrial metabolism and its intracellular number. Other studies reported only a few cases with inflammatory breast changes. Palmedo et al. [46] reported two false-positive results in three women with benign inflammatory breast lesions. One study reported the characteristics of MIBI scintimammography in acute mastitis and found that in this pathologic condition, MIBI tends to concentrate and accumulate in areas of active mastitis and inflammatory breast lesions [47] but becomes normal after successful treatment of the infection.

Up to 20% of all cancers arise in association with chronic inflammation and most, if not all, solid tumours contain inflammatory infiltrates. Immune cells have a broad impact on tumour initiation, growth and progression, and many of these effects are mediated by proinflammatory cytokines. Among these cytokines, the pro-tumourigenic functions of TNF and interleukin 6 (IL-6) are well established. The role of TNF and IL-6 as master regulators of tumour-associated inflammation and tumourigenesis makes them attractive targets for adjuvant treatment in cancer [48].

In this study, the results showed that the *in vitro* and *in vivo* mRNA expression by RT-PCR after standardising the techniques was similar. Our results reported that asiaticoside has an effect on cytokinin expression in DMBA-mediated tumours and MCF-7 cells. Asiaticoside decreased the expression of tumour necrosis factor-alpha (TNF-α), a cytokine involved in systemic inflammation that is a member of a group of cytokines that stimulate the acute phase reaction. The primary role of TNF lies in the regulation of immune cells. TNF is an endogenous pyrogen, which is able to induce fever and apoptotic cell death and sepsis. This protein is chiefly produced by activated macrophages although it can be produced by other cell types as well. In addition, the results showed that asiaticoside decreased the expression of interleukin-1 beta (IL-1β). IL-1β is a member of the interleukin 1 cytokine family. This cytokine is produced by activated macrophages as a proprotein, which is proteolytically processed to its active form by caspase 1 (CASP1/ICE). This cytokine is an important mediator of the inflammatory response, and it is involved in a variety of cellular activities, including cell proliferation, differentiation, and apoptosis. Here, asiaticoside decreased the activity of cytokines that are important mediators of the inflammatory response and are involved in cellular proliferation, differentiation, and apoptosis.

Our results are in agreement with many studies that have reported that asiaticoside has anti-inflammatory activities in several inflammatory models. Asiaticoside has protective effects against sepsis-induced acute kidney injury, which is most likely associated with the inhibition of IL-6 in serum and the iNOS protein in kidney tissues [49]. Asiaticoside reduced the content of IL-6 and TNF-alpha in a dose-dependent manner in acute lung injury [50,51].

In this study, asiaticoside suppressed proliferation, decreased MIBI uptake and diminished the growth rate of *in vivo* 7,12 dimethyl benzanthracene (DMBA)-induced mammary tumours in rats and *in vitro* MCF-7 cell uptake; however, the mechanisms for these process remains unknown. There are many different mechanisms through which asiaticoside can act in cancers and other tissues. For example, asiaticoside inhibits hypertrophic scar fibroblast formation from the S to M phase through the Smad signalling pathway [52]. Asiaticoside possesses good wound-healing activities in many species, including humans, because of its simulative effect on collagen synthesis [53] and the reticuloendothelial system [54,55], which relieves inflammation. Asiaticoside promotes apoptosis and alters cell membranes by an unknown immune-mediated mechanism. Overall, the results of this study showed that the administration of asiaticoside significantly reduced the percent of tumour growth that is significantly correlated with MIBI uptake ratios, and this is also correlated with caspase-3, TNF-α and IL-1β values.

## Conclusion

The results of this study suggest that asiaticoside acts as a biochemical modulator that induces apoptosis *in vitro* and *in vivo* in MCF-7 cells and DMBA-induced rat cancers, respectively. Asiaticoside has potential chemopreventive, antitoxic-enhancing anti-tumour activity, and anti-inflammatory effects.

Asiaticoside significantly reduces *in vitro* and *in vivo* tumour volumes. After a course of asiaticoside treatment, all rats that developed multiple mammary tumours exhibited tumour regression and reduction. In addition, asiaticoside had significantly reduced the TNF-α and IL-1β cytokinin and MIBI uptake ratios. The causes and mechanisms of prevention require further investigation. Future studies should be performed to confirm our findings and further delineate the clinical role of asiaticoside.

### Declaration

This is to declare that the experiments comply with the current laws of Kuwait where they were performed.

## Abbreviations

DMBA: 7,12-Dimethylbenz(a)anthracene; MCF-7 cells: Human breast adenocarcinoma; MDA-231 cells: Human breast cancer; HBL-100 cells: Human breast, epithelial carcinoma; PC-3 cells: Human prostate cancer; HaCaT cells: Human keratinocyte skin; pII: Oestrogen receptor down-regulated transfected cell line derived from MCF-7 cells; MTT: 3-(4–5 dimethylthiozol-2-yl)-2,5 diphenyl-tetrazolium bromide; TNF-α: Tumour necrosis factor-alpha; IL-1: Interleukin-1; MIBI: Technetium-99 m hexakis-2-methoxyisobutylisonitrile, Technetium-99 m-sestamibi, ^99m^Tc-MIBI; IC50: 50% inhibitory concentration; TcO-4: Pertechnetate; Mo-^99m^Tc: Molybdenum-99-technetium-99 m; Advanced DMEM: Advanced Dulbecco’s Modified Eagle; FCS: Foetal calf serum; WT: Wild type; DMSO: Dimethyl sulphoxide; S phase: DNA synthesis phase; PBS: Phosphate Buff Saline; ELISA: Enzyme-linked immunosorbent assay; FBS: Foetal Bovine Serum; PI3K: Phosphoinositide-3 kinase; Bax: Bak, Bad, Bcl-Xs, Bid, Bik, Bim and Hrk, Bcl2 and Bcl2 family proteins; TRAIL-R1 and -R2: TRAIL receptor-1 and 2; DR3: DR4 and DR5, Death receptor 3, 4, and 5; PAF: platelet activating factor; COX1 and COX2: Cyclooxygenase 1 and 2; RT-PCR: Reverse transcription polymerase chain reaction; ip: Intraperitoneally; HI: Weight homogeneity index; Wl: The lowest weight; Wh: The highest weight; W_g_: Weight gain; W_0_: Weight recorded in the beginning; W_x_: Weight recorded in the end; V: Volume of tumour; a: The longest diameter of a tumour; b: The shortest diameter of a tumour; MBq: Megabecquerel; KeV: Kiloelectron volt; ROI: Region of interest; WB: Whole body; H&E: Hematoxylin and eosin; SD: Standard deviation; ANOVA: One-way analysis of variance; SEM: Scanning electron microscopy.

## Competing interests

The author declares that there is no financial relationship with Kuwait University that has sponsored the research and no conflict of interest.

## Authors’ contributions

FA has made substantive intellectual contribution to this study, and the conception, design, acquisition, analysis and interpretation of its data. FA drafted the manuscript, critically revised it for important intellectual content, and has approved the final version.

## Pre-publication history

The pre-publication history for this paper can be accessed here:

http://www.biomedcentral.com/1471-2407/14/220/prepub
